# The contribution of a complex systems-based approach to progressive social resilience

**DOI:** 10.1177/13634593231195784

**Published:** 2023-08-30

**Authors:** Philip Haynes, Angie Hart, Suna Eryigit-Madzwamuse, Matthew Wood, Josie Maitland, Josh Cameron

**Affiliations:** University of Brighton, UK; University of Brighton, UK; University of Chichester, UK; University of Brighton, UK

**Keywords:** complex systems, resilience, social resilience

## Abstract

The use of resilience in social practice has evolved from a theoretical framework at the intersection between individuals and their social ecology. Critics argue this theory still results in policies and practices that are too individualised, with the potential for negative social consequences. This paper further critiques contemporary understanding of resilience theory and its application. It juxtaposes complex systems theory with a social inequalities oriented resilience practice. This provides a paradoxical approach. It is acknowledged that state and public policy decisions and actions can be anti-resilient, undermining community and social resilience that already exists in the form of social relationships, self-organisation and co-production. Nevertheless, collective social resilience also illustrates the potential of local and service user organisations to contribute to an overall transformational change process.

## Introduction

This article aims to combine a complex systems-based theoretical framework with progressive approaches to social resilience. We advance this juxtaposing with reference to recent updates to resilience and systems theory. In resilience theory and practice, this includes the entanglement of social factors with individual factors and the normative imperative to understand social inequality as a contributor to adversity (Hart et al., 2016). This leads to an explicit focus on the necessity for co-production when creating social and psychosocial interventions.

Complexity theory has a growing influence on applied social science. It explains social systems as dynamic and unpredictable, and often in conflict with each other. When the concepts of resilience and complex systems are understood and combined there is evidence of the potential to develop radical and community-based approaches to resilience practice. We argue that this theoretical framework avoids the pitfalls of assuming that social systems normally have some positive social functions where systems are often resilient in their adaptations to achieve public value. Instead, the contemporary complex systems approach evidences a realisation that public policy and unregulated market system actions can actively undermine social resilience and cause social dynamics that are ‘anti-resilient’, by increasing social inequality, personal isolation and mental health difficulties. The authors draw on their experience of community mental health and wellbeing in this respect.

We first define an overview of contemporary issues in resilience theory and social systems and their attempts to deal with resilience theory as individualising and neoliberal. Second, we summarise the evolution of systems theory into complex adaptive systems (CAS) and into social science, and the resulting explanations. These are approaches that draw on macro social and relational influences, and include the structural causes of disadvantage and inequality (Walby, 2007). Finally, we infuse current resilience theory with a richer conceptualisation of complex systems where social systems are highly unstable and often in conflict with each other in a manner that undermines resilience. This is a dynamic we refer to as ‘anti-resilient’. We seek to give a social and progressive lens on resilience that includes explicit recognition of the reasons for social injustice and recognition of system and structural forces that can undermine resilience.

The concept of resilience has had a growing importance in a diverse range of subject areas including ecology (Lebel et al., 2006), political economy (Hall and Lamont, 2013), developmental psychology (Masten, 2016), social work (Ungar, 2013), social marketing (Wood, 2016a, 2016b), business and organisational management (Burnard and Bhamra, 2011), child and family therapy (Hart et al., 2007), public health (Meyer et al., 2018) and social policy (Dagdeviren and Donoghue, 2019). Fraccascia et al. (2018) calls for each field to recognise the main aspects that are important to their specific research and practice domain.

Resilience has been a topic of interest for developmental psychology since it was found that early adversity might increase the risk of adverse biopsychosocial developmental outcomes. Resilience is, therefore, doing better than expected under conditions of high risk. The variation in outcomes led to the contemporary evolution of resilience theory as a framework for developing interventions. Initially research focused on factors considered more internal to the individual. This individual focus has developed into the study of epigenetic changes and stress-regulation systems in neurobiology (McEwen et al., 2015). External, social influences, are also included in contemporary approaches to resilience.

For example, the socio-ecological model developed by Bronfenbrenner (1979, 2005) and applied by Ungar (2013), states that to build resilience, the primary focus should not be solely on understanding characteristics of individuals, but rather how they navigate their socio-ecological environment and negotiate protective factors. How do the multiple systems that individuals interact with facilitate them to develop resilience or not? Due to an emphasis on person-environment interaction, and a need to reconcile this with social influences, many conceptualisations of resilience are becoming systems orientated (Roisman et al., 2002) including those applied to practice (Barasa et al., 2017; Kieny et al., 2014; Meyer et al., 2018; Porroche-Escudero et al., 2017; van de Pas, 2017).

Current resilience based practices in psychosocial interventions commonly have two main critiques. Firstly, a concern that current resilience practices are still too individualised (Bottrell, 2009; Friedli, 2012; Harrison, 2012) and resilience is theorised as ‘agent centric conceptualisations’ (Dagdeviren et al., 2015: 1). And secondly that this individualism leads to problems with the values base of resilience approaches because they can negate social responsibility and justice, and are likely to implicitly support neo-liberal ideology (de Lint and Chazal, 2013; Garrett, 2015; Hart et al., 2016; Joseph, 2013). Any theory applied to humans and their social environment must consider the values, value conflicts, subjective meanings and perceptions and inherent power imbalances (Garratt et al., 2021). Neocleous (2013) argues that the problem with resilience is that it is by definition ‘against resistance’. He takes a holistic and structural approach to the various previous criticisms of the concept of resilience. Neocleous concludes that the whole resilience theoretical discourse is opposed to the idea of resistance when communities face anti-social and state backed politically oppressive forces that seek to break their social identity and relations. This argument requires a critical and transformative approach to system based understanding of resilience that adds further dimensions of complexity to resilience and its implications. The application of complex systems theory to the concept of resilience is the way forward here. It makes the distinction between the broad umbrella of systems theory that resilience theories have originally embraced, and the specifics of complex systems theory, where systems are unpredictable, dynamic, unstable and often in conflict with each other, as articulated in this paper.

Despite their complexity, complex systems can be compared and understood in comparative terms, because they have similar (not identical) patterns and processes (Haynes, 2017). We argue that theorising resilience with this complex systems ontology results in seeing some evidence of social resilience, like shared and complimentary values, relationships and optimal communications of person-environment interaction, but that these system qualities of resilience are not evident all the time, nor are apparent in all systems. The additional part of the complex systems analysis is that externalities, and systems in conflict with each other, can have impacts that undermine and damage the resilient component of any one particular system. In this way, one system can cause another to be anti-resilient. Complex social systems include conflicting structures of competing values and beliefs (Haynes, 2018). Resilience approaches in psychosocial interventions need to be placed explicitly in a values context. In such an approach, research should include a political and power based analysis of identifying who is subject to adversity, and why. Solutions should be co-produced with the aim of changing social structures to deal with the causes of the adversity (Hart et al., 2016). Dagdeviren et al. (2015) and Dagdeviren and Donoghue (2019) argue this is essential when understanding the causes of social disadvantage and its negative impact on mental wellbeing. We argue that resilience and complex systems theory combined can recognise conflict and power imbalances which are the largest underlying causes of social disadvantage and negated wellbeing and resilience.

### Resilience theory and social systems

In the next section, we outline the development of a social systems approach to resilience. Roisman et al. (2002: 1216) summarise this as:

Resilience is an emergent property of a hierarchically organized set of protective systems that cumulatively buffer the effects of adversity and can therefore rarely, if ever, be regarded as an intrinsic property of individuals.

### Social interactions and communications

Social systems are largely defined by their connections, across collective groupings and between individuals (Luhmann, 1995). These connections do more than the sharing of information – they also generate and reinforce shared values and behaviour. Social interactions have the potential to create collective resilience. Complex social system structures and dynamics, however, include multiple overlapping systems and boundaries and powerful controls and behaviours. These can impact resilience in some system places and damage resilient social infrastructures. For example, social isolation is associated with poor mental health (Kawachi and Berkman, 2001). State actions and public policies like the ending of support for industries and reducing income benefits can contribute to isolation and a decline in wellbeing. Families living in low-economic households often experience less support with high levels of stress and decreased access to resources (Balaji et al., 2007; Morris et al., 2017). Inequality can undermine positive social connections and the ability to prosper in social systems.

In contrast, social support builds connections, reduces negative impacts and directly enables wellbeing and can contribute to resilience (McConnell et al., 2011). The development of supporting connections can facilitate collective ‘social resilience’, defined as the: ‘capacity of groups to sustain their well-being’ (Hall and Lamont, 2013: 22). There is no guarantee that such collective responses can determine a transformation of structural disadvantages, as demonstrated by research about how people coped with hardship after the great recession of 2008 (Dagdeviren and Donoghue, 2019). Nevertheless, the process of empowering connections creates opportunities for seeing inequalities as having structural causes and this may enhance mental wellbeing and the shared belief that collectively driven change is possible. Policies should therefore seek to empower neighbourhood and community co-produced services and outcomes.

Social resilience-based approaches entail understanding the interactions and communications between individuals, families, service providers and the wider community. Then, applying this understanding to offer appropriate support, through holistic policy programmes that redistribute resources and are co-ordinated across multiple system levels (Hart and Heaver, 2013). There has been criticism of the lack of such a coordinated approach to mental health policy in the United Kingdom, with the resulting fragmentation and danger that more specialist and intensive services are needed to assist when preventative and first response services are reduced (Haynes and Stroud, 2019; National Audit Office, 2023).

Social resilience aims to ‘scale up’, to such an extent that it can influence key connectors and power brokers in the macro systems of relevance. The emergence of scaling is a key feature of the growth of social movements and how they can transform injustices, like racism, poverty and homelessness that cause poor mental health. As Chesters and Welsh (2005: 192) noted, there are: ‘two key processes in the emergence of . . .social movements, the process of encounter and interaction and the process of constructing shared understandings’. The relationships that are constructing resilience therefore become a form of resistance, a social and political movement that focuses collective energies against structures and social injustices that undermine mental wellbeing. Resistance to anti-resilience policies assists the maintenance and rebuilding of resilience and seeks to acquire state resources and policy actions that restore the positive social constituents of social systems. An example is resistance to the over-use of mental health legislation to detain those from ethnic minorities who are experiencing mental health difficulties, a trend that is associated with the multiple social disadvantages for these communities and the removal of resources in socially supportive community-based facilities like day centres and similar (Haynes and Stroud, 2019; Wessely et al., 2018).

### The system dynamics of risk and protection

Two concepts that have featured in previous resilience literature are: ‘protective’ and ‘risk factors’ (Este et al., 2009). At the community level, it has been argued that resilient communities have the capacity to provide a network of protective services and support when things go wrong, for example, a natural disaster (Ungar, 2015; Wright et al., 2013). These protective capacities include caring and supportive relationships, collective identification with a common goal, a shared compassionate culture, equitable access to resources and spiritual leadership (Alawiyah et al., 2011). This is a dynamic social environment, where resilience interacts with significant risks associated with adversity conditions and their impact on wellbeing. Much adversity results from social structural factors that can impact the mental health of individuals. Factors related to inequalities such as poor housing, lack of educational opportunities and poverty have been shown to predict multiple risks to mental health (Wilkinson and Pickett, 2010). These structural factors undermine community resilience and show how the state and its policies can be anti-resilience.

Resilience practices are at risk from structural changes and their consequences, such as the closure of mental health wellbeing day centres and specialist supportive accommodation that are not part of statutory services. This is when a community that has built and supported caring relationships has its efforts undermined in an anti-resilient decision by the state such as reducing funding grants to community provided resources (Ungar, 2015; Wood and Shukla, 2019). This shows how the dynamic process of building social relations can be undermine by financial crisis ‘austerity’ public expenditure cuts and their implications for the provision of local services.

Major social transformations, like reductions in depression and anxiety, involve changing the structural causes of adversity. In a supportive policy environment, resilience practices can identify the characteristics and mechanisms that are potentially protective (Este et al., 2009). This will only happen if local level collective activity is supported to scale up and is encouraged by government to take its part in achieving macro structural change that is locally relevant.

### Social capital

The potential to build social resilience from collective relationships can be linked to Bourdieu’s (1986) notion of ‘social capital’. The assets that people have to protect themselves from social hardships are not merely economic, in the form of stores of wealth and income, but also take the form of social capital acquired through their experience of social connections, and ‘cultural capital’ that includes assets such as education and knowledge and experience of culture. Cultural capital is also dependent on state interventions to spread opportunities to all, beyond the ownership and purchases of the most wealthy and powerful.

Social support can be defined by both its functional and structural components. The functional components are the emotional (e.g. comfort, empathy), instrumental (e.g. assistance with material needs) or informational assistance (e.g. personal advice) that others provide (Letourneau et al., 2004). Social networks are the structural component of social support. This includes the number and type of social connections with individuals and community. For Kawachi and Berkman (2001), it is the collective value of these social networks (e.g. having someone you can trust or rely on) that grows social capital. These elements of social support can be uniquely important in reducing stress and promoting better mental health outcomes for low-income families (Luo et al., 2012). The development of functional and structural components therefore work in a dynamic and relational complex social environment, but this also requires a supportive governmental and economic system that provides positive reinforcement, including resources.

### Social resilience

A fundamental role in developing the process of resilience and the capability to recover and transform circumstances is played by the quality and the spread of social and community networks. After a natural disaster, like the COVID-19 pandemic, improved social connectivity corresponding to higher levels of social capital may turn out to be as important as other factors such as resources from government. This is due to the solidarity and altruism spread in a community that facilitates cooperative action after an external shock. On the other hand, neighbourhoods with a low level of social capital in the event of a disaster are less able to work cooperatively and to have the prior knowledge and power base to ask for assistance from public authorities (Aldrich and Meyer, 2015; Aldrich and Smith, 2015). Although communities need resources in adverse situations, they also need to be able to participate and provide local expertise about the use of these resources. As Hart et al. (2016) has argued, resilience needs to facilitate emancipatory elements like activism and advocacy. Resilience is political and participatory. Hall and Lamont (2013: 14) comment that: ‘social resilience is the result of active processes of response’. Resilience promotes ‘transformation over an earlier state’ (Hall and Lamont, 2013: 13), rather than passive coping with an undesirable and unjust situation. The issue is not only how an individual withstands an unjust situation (Donoghue and Edmiston, 2020), but how a social group can comprehend progressive change in relation to its causes. In a society composed of numerous dynamic and sometimes conflicting social systems, access to the political process to raise agenda items and influence policy and practice implementations is essential to maintain community resilience. Our paper locates this social and transformative approach to resilience in the context of a dynamic and realist version of complex systems (Byrne, 2011). Systems are not value neutral and returning to a static version of economic and political equilibrium, but are inherently conflicted and unstable with limited periods of stability and predictability that need political management and a value based vision. Resilience exists in this dynamic and is not automatically self-generated. It depends on the evolution of supportive and socially just social structures and policies.

### A systems approach to resilience

Historically, systems theory was first juxtaposed with resilience in the field of ecology (Holling, 1973). In social science, a resilience systems approach has developed which aims to embrace systems that include different levels of social phenomena. The key levels are: the individual (micro), the organisation (meso) and the nation state (macro). Resilience theory therefore acknowledges multi-level influences on resilience across hierarchical levels (Carvalho et al., 2012; Sutcliffe and Vogus, 2003).

A systems-based approach to resilience emphasises the impact of both individual and social environmental factors on mental wellbeing (Bronfenbrenner, 1979, 2005; Wood, 2016a, 2016b). In some systems interpretations of resilience, those to which our argument is sympathetic, resilience emerges from the system factors, rather than from within the individual.

In advocating a socio-ecological approach to resilience, Ungar (2015: 6) argues: ‘change is unsustainable without access to an environment that supports the client’s process of growth’. It is, therefore, argued that changes to social ecologies are likely to have a greater impact at scale on mental wellbeing than interventions focused on individuals (Prilleltensky, 2012).

Holling’s (1973) seminal account from which the social ecological approach has grown was a conceptualisation of a natural science ecosystem. Any system applied to humans and their environment must also take into account the metaphysical properties of values, beliefs and perceptions. This potential metaphysical void, where facts based on evidence are separated from value based perspectives, is a major concern when translating scientific theory into the social realm (Gerring and Yesnowitz, 2006: 38). Values, beliefs, perceptions can be unpredictable and change with context and time. Furthermore, changing values can shape and reorganise the system. This adds to the complexity in a system that already involves multiple layers of interaction. Complexity theory exposes these unstable dynamics and their importance in the realities of the conflicted social, political and economic world.

An analysis of systems resilience and who benefits always needs to be located in a political and participatory framework that facilitates and learns from user perspectives (Hart et al., 2016: 6–7). Solutions should be co-produced with those who need the solutions.

This interest in building social resilience is also increasingly applied to organisations (Burnard and Bhamra, 2011) and even nation states (Hall and Lamont, 2013). For example, with regard to organisations, public health approaches are noticably interested in the ability of health organisations to be resilient to crisis based demands (Kieny et al., 2014). These demands can be understood as part of the macro social system, caused by systemic factors, and malleable to change as a result of political processes. Therefore, some resilience theorists (Hart et al., 2016) have argued that resilience responses can and should involve modifying or transforming systemic adversities like social stigma towards mental health problems. This is the notion that resilience is: ‘overcoming adversity, whilst also potentially changing, or even dramatically transforming, (aspects of) that adversity’ (Hart et al., 2016: 3). This definition of resilience implies a complex systems perspective, in which the relationship between individuals and wider social forces is complex and often trapped in hierarchical power relations across the macro, meso and micro levels (Walby, 2007).

The additional implication that a complexity theory approach to system resilience needs to add to the previous systems analysis is that some systems can work to undermine the resilience of others. The global economic liberalisation since the 1980s has undermine the resilience of many traditional working-class communities. It has brought the precarity of low-income jobs and a constantly changing employment market. These social aspects have made citizens more vulnerable to inequalities, poverty and mental health difficulties. Resilience is not a universal, guaranteed attribute of all systems. Rather it is a dynamic relational process, subject to periods of disruption and damage, in addition to occasions of opportunities for development and growth. This is similar to the operation of anti-resilience where the ability of systems to be resilient, especially at the micro and community level, is undermined by other systems and their processes.

## Social complexity as a systems perspective

Complex systems theory is a grand narrative for understanding society as a network of entangled and relational social systems. It has had a growing influence on applied social science in the last two decades (Byrne, 1998, 2011, Byrne and Callaghan, 2023). Historical influences include both chaos and complexity theory in the natural sciences, especially scientific research that informs weather forecasting (Lorenz, 1963), but which also has influence across a range of disciplines (Simon, 1962). More recently, it has been proposed that mental health policy can be theorised as a complex dynamic system (Haynes and Stroud, 2019; Kuranova et al., 2020).

Complex systems theory emphasises the highly dynamic interactions and relations in social systems. Because of the complexity of interactions, a science of predictability based on historical evidence is problematic. In the social relations of these systems, causal mechanisms are unstable over time; they are heavily dependent on contexts, externalities and the specific place of operation. For example, a model that predicts the spread of a global virus like COVID-19 will have limited use. The spread will vary in different countries and localities, because of different social networks, cultures, age profiles and policy interventions (Thacker, 1986). Structural change is difficult and much stands in the way to prevent it, but change is possible, especially at the points in time when systems and their interconnections are relatively unstable (Snowden and Boone, 2007).

The challenges of these complexities have important lessons for social interventions. The phenomena of social complexity means that practitioners need a humility in their practice, because much is unknown (Berger, 2019). Research evidence and its influence on future interventions is never guaranteed to be completely successful (Etzioni, 2014). This is not an argument for the redundancy of social science theory and research, but an argument for realism about their use.

## Defining complexity

One of the best articulations of the conceptualisation of social complexity came from the late Cilliers (1998: 3–4). What follows is a paraphrase of his seminal definition.

### Cases

Complex social systems are populated by cases, that is people (actors), organisations and countries. Cilliers’ focus on cases can be further understood by examining the social science definition of cases given by international experts in case-based methods (Ragin and Becker, 1992), and specifically, Berg-Schlosser et al. (2009: 6). The latter define a case as: ‘a complex combination of properties, a specific whole that should not be lost or obscured in the course of the analysis’. This has similarity to Archer’s (1995) idea of *morphogenesis* as the form and shape of a living system. It is immediately apparent that cases are situated at overlapping levels: people, organisations, countries. Cases in social systems are also essentially complex systems themselves (Gerrits, 2008). Cases are largely defined by complex interactions with other cases. These interactions occur vertically, for example individuals communicating with groups in an organisation, or horizontally, for example an individual communicates with another individual. Most contacts people have with others are local, or nearby, even in the internet age where much digital communication has been shown to still be taking place in the locality. An individual’s mental wellbeing is dynamically interconnected with their local economy and community and these social aspects are linked with mental health (Haynes and Stroud, 2019) The juxtaposition of complex systems and resilience confirms that to understand resilience and mental wellbeing requires an understanding of the local social systems context and what services are provided or unavailable (Table 1).

**Table 1.** A juxtaposition of complex systems and progressive resilience theory.

Ideas/concepts from complexity theory	Understanding social systems	Understanding resilience	Using a complex systems lens to understand resilience
Cases (i.e. people, organisations, countries)	Social systems are constructed from social interactions in the midst of complexity and instability.	Resilience evolves across case levels – people organisations, and nations.	An individual case is not alone, but always exists in a complex social context.
Elements: Physical (i.e. buildings, computer networks) Metaphysical (i.e. beliefs and values)	The relationship between cases and elements is defined in processes that are dynamic, temporal and precarious.	Elements have a dynamic interaction with external social change and the adapting needs of people.	Social processes include conflicts and instability. They require adaptive responses from policy and organisations. Processes cannot be overly rigid or bureaucratic in their controls.
Interaction	Social interaction is at the core of complex systems and giving purpose to dynamic and unstable systems.	Resilience is a form and quality of social interactions underpinned by appropriate resources.	The quality of interactions in the context of system instability determines the experience of resilience and can include collective resistance.
Communication and Feedback	Complex systems are defined by communication and feedback that ‘reinforces’ or ‘negates’ and has impact on behaviour.	The quality of system interaction is dependent on good communication and understanding, so cases can take appropriate action.	Social resilience is underpinned by good communication, shared values and supportive external resources.
Self-organisation	Historical structures and patterns tend to dominate social systems, but not exclusively so. Innovation is possible from the bottom up, with opportunities for tipping points and transformations.	Resilience can emerge from small groups, neighbourhoods, communities, in collaboration – against the odds – and through shared values and purpose. It can include resistance.	The importance of community based relationships and communication, to resist negative changes, and for the emergence of progressive ideas.
Patterns	Complex systems are not mechanistic or easy to predict in causal ways, but they can be partially understood as patterns of similarity that may get repeated.	Social resilience is patterns of interactions and community development that includes political resistance.	Sharing communal stories of systems events and formulating responses that aim to improve collective wellbeing.
Interventions	Interventions need to be sensitive to local needs and adaptations, if they are to support and nurture ‘social progress’.	Interventions that build a coproduced ‘people process’ and have adequate and appropriate resources are more likely to be resilient and enduring.	The importance of a supportive political and policy process, that establishes a social network with a vision for change and control over local resources.

### Elements

Social systems have different elements, for example, the material resources used by people and organisations, like health service buildings, and pharmaceuticals. The relationship between cases and elements lies at the heart of conventional approaches to social systems management, especially with regard to organisational process and production. Social systems, however, also have metaphysical, socially constructed elements, such as values, beliefs, cognitions and cultures. Human actors spend much time giving meaning to the operations of their social systems. This activity is networked and intertwined with other systems. For example, the mental health system derives meaning for diagnosis and treatment, and this has to be related to the needs for learning in other systems, such as, the legal system, political democracy, economics and the family. Luhmann (1995) described these system connections as ‘structural couplings’ as they require certain formal communication places, connectors and processes, in order to be able to communicate with each other.

Haynes (2018) proposed that complex social systems are comprised of entanglements of values that operate both hierarchically and vertically across society. This results in cognitive complexity for the individual actor who has to simplify these challenges in any one time and space to make a pragmatic decision. As a consequence, this may include them resolving cognitive dissonance in their own values and their application to decisions. A similar resolution process happens at the meso and macro levels. Kontopoulos (1998) has argued that there are dominant social values (he calls them ‘totalising logics’) that have a long-standing influence over time and space on society and nations. The dominant medical model of mental health can be viewed in this way. Political conflict becomes an inevitable part of social systems and their change, and conflict resolution is a key activity. Resilience and whether it occurs or not in any one system is dynamic and changing because of these conflicts.

Given the dynamic nature of society and the local context within which much social systems activity is located, the meso and macro elements in complex systems are fragile and prone to change. Resilience can decline or it can increase, dependent on a range of complex interactions. System resilience and the ability of people, organisations and nations to be resilient relies on adequate external supports and adaptations, including those provided by other social systems. To survive and prosper requires system adaptation. These adaptations are therefore a key association between complex systems and resilience (Table 1).

### Information, knowledge and communication

Communication is the lifeblood of social systems. Without communication, systems cannot exist and function (Luhmann, 1995). This is the social network that forms a psychosocial fabric to any human system. The communication flow of information about a complex social system has major implications for its future trajectory. There is no ‘end point’ or ‘sum of all information’ about the system, because there are different perspectives from different cases, not one single perspective. No actor or organisation – or national state – has perfect information, but only a partial and limited view. Information is processed into knowledge in the form of theories, heuristics and stories about the working of the system and how it can be used, managed and directed. Like information, no knowledge is ever fully complete, and knowledge about the system is – like the system itself – dynamic and evolving (Cilliers, 2005). Given the dispersed nature of information and knowledge there is a limited ability of structures to create dominance and stability and there is also a potential for instability and change. In the context of social intervention to build resilience, this leaves room for agency and change. Approaches to mental wellbeing do adapt and change but for a complex variety of reasons. System resilience is therefore a highly dynamic relational process in this respect.

For cases to have resilience within social systems, the quality of their communication becomes key. Communication alone is not enough for cases to survive and thrive, communication of quality must build purposeful knowledge and relationships, to enable collaboration and the continued sharing of useful information. In doing so, communication builds the underpinning shared values for those experiencing the system and gives them a collaborative perspective (Chesters and Welsh, 2005). Through shared values it can extend across social systems and provide new and priority resources. While complex systems theory focuses on the importance of communication to a system, resilience theory indicates that this communication needs to be of a useful quality and able to confirm shared values and inspire collaborative behaviour, if resilience is to flourish and protect mental wellbeing (Table 1).

### Self-organisation

Self-organisation in complex systems theory is the emergence of ideas, communication and behaviour from the micro level. Olsson et al. (2015) are critical of the fact that self-organisation can be presented as a ‘universal truth’ that always aids system adaptation and resilience, but this ignores a developing nuanced approach to self-organisation in social research (Teisman et al., 2009). Conservative self-organisation (preserving the system state, via local communication and behaviour, to keep things as they are) can be argued by different perspectives to be either ethically good or bad. Conservative self-organisation needs to be viewed as a plurality and cannot be assumed to be universally good.

Likewise, dissipative self-organisation, that is local micro innovation to enable a system to change and adapt from the ‘bottom up’, can also be argued by different parties to be either ethically good or bad, for different reasons. Local innovations might be argued to be contravening social justice and equality in some circumstances. It is the specific social justice context of these examples, and a critical analysis of who benefits and who loses from change, that illuminates the normative nature of social relations and social conflict. We, therefore, argue that systems concepts can be useful tools for analysing complex social processes, but while using them to recognise and expose the normative interpretations of systems change (see Table 1).

In complex social systems theory, self-organisation illustrates that while social structures regularly give some powerful groups control over others, agency and small scale, bottom up change is possible. The link here with resilience theory is the importance of small-scale collaboration and support and resistance against other social forces (Shakespeare, 2006). It is possible that resilience might provide resistance in some circumstances, rather than being the antithesis of it (Neocleous, 2013). In combination, this suggests that much progressive social change will start small scale and need nurturing, before it can be scaled up to have a bigger impact (Table 1). Resilience might include the conservative, protective defence of existing civil rights and resources through resistance to change. For example, in the history of mental health provision in England, like trying to protect hard won rights for service users subject to mental health legislation, or preventing a local voluntary organisation day care centre from being shut because of a lack of funding. The history of changes to the mental health system in England, shows that both conservative social organising to prevent negative change and dissipative, innovative organising to argue for new and positive changes can be important to enhance mental wellbeing.

### Patterns

Complexity theory puts emphasis on the presence of instability in systems rather than assuming system stability (Urry, 2005). This is the subject of some controversy. Original physical systems scholarship in the natural sciences looked for mechanistic and interactive terms that could explain relative stable states. Instead, the complex adaptive systems perspective puts emphasis on ‘dynamics’ (Haynes, 2017), where the causal mechanisms that do exist are at best partial and often limited to a context. Micro interactions create patterns that while similar, are rarely identical. The spread of disease, like COVID-19 and its impact on mental wellbeing in different local communities, creates such patterns. What stability that does exist within complex systems can best be described as patterns, but patterns that will evolve and change over time. The degree of instability may be small, so an evolving pattern continues to be recognisable, even though it does not remain identical. Weather clouds are a good example of such a phenomenon in the natural sciences. We recognise a cumulus shape and colour, but never see two identical clouds. In the social realm, forms of behaviour demonstrated by vulnerable social groups, like loneliness, isolation, depression and anxiety, take on recognisable and repetitive patterns, and these are evidence of public health trends, but they are never identical at the micro level, and often evolving to a different dynamic state. Similarly, macro level structural changes like the dilution of civil rights, cuts to public expenditure and services, can be recognised for their negative impact on mental wellbeing, but experienced in slightly different ways in localities in terms of the degree and scale of how they impact local people and services.

While complex social systems are indeterminate and follow many possible future trajectories, these trajectories have a probability of falling within certain boundaries, and forecasts can speculate on the most likely of trajectories. Public health planners do better at thinking about a range of scenarios rather than trying to be precise about future trends. This also has important consequences for the deployment of resources. Resources and responses need to be adaptable to local needs and context, rather than modelled too rigidly to one manifestation of behaviour and how to intervene in it (Haynes, 2015).

Complexity theory seeks to develop a scientific reasoning for codifying when the evolving of patterns becomes more substantial and significant. This is when a fundamental social pattern breaks down and a new category of pattern comes into existence. Some have referred to this major changing of patterns as a ‘tipping point’ (Byrne, 1998). For example, mental health trends in communities can evidence a key period of change, such as the COVID-19 pandemic, when certain demographics are more vulnerable in a way that results in practitioners having to readjust their priorities and interventions. One cannot assume that resilience will instinctively be available as a community resource in such crises, because the overall system dynamics might also undermine and damage the ability to ‘be resilient’. This illustrates the high degree of unpredictability within complex systems interactions.

For complex social systems theorists, understanding comes more often from perceiving patterns in relation to a given spatial and temporal location, rather than discovering universal and generalisable mechanisms. We can juxtapose this with resilience theory by noting that for those seeking to be resilient, similarities exist in their experiences of society in the local setting, and it is the collaborative sharing of these experiences that potentially helps to build local resilience, and the opportunity to initiate positive change at the structural and macro level (Table 1). Similarly, external factors like new resources might empower and energise manifestations of resilience from within the local system. Resilience emerges and/or fails in the social, rather than from within the individual. It is best understood by observing patterns of social similarity, rather than expecting mechanistic and generalizable rules to be applied to individuals.

## Interventions

System based approaches usually start by stakeholders spending some time learning about how a given system really behaves, from a number of perspectives, thereby increasing one’s understanding of the current complex system, before making a difficult judgement about how to intervene (Seddon, 2008; Snowden and Boone, 2007). A recent example of such an approach is the ESRC CECAN programme’s use of Participatory Systems Mapping (Penn and Barbrook, 2019). Such a method may take time and will most likely expose differences and conflicts about the purpose and priorities of the system. It should strengthen collective understanding and a value-based commitment to what needs to change. This method can be used to better understand the social experience of regional and local mental wellbeing (Watts et al., 2020). Analysis of the resulting participatory map can lead to a sharper focus on the relationship of the system with the environment and an informed strategy about how to achieve change. These participatory interventions need to be sensitive to local needs and adaptations. Interventions that are proposed to change the system are unlikely to be quick fix mechanisms. The process of systems participation adds value to the intervention by the sharing of information and improving the quality of communication (Table 1). It generates new collective knowledge. Examples in mental health policy are the multiagency activities of local council joint needs assessment and plans that seek to work with the National Health Service, communities and service users to best understand the nature of wellbeing in a local area and how resources can best be allocated to improve mental health.

The late environmental activist and thinker, Meadows (2009), whose thinking and vision of sustainability was ahead of the political age she lived in, concluded from applying such systems approaches to interventions that the best chance of achieving system transformation, was to identify how to adjust values and change the overarching paradigm. In the current policy debates about improving mental health and wellbeing, there is a sense that major change can only be achieved if social acceptance and understanding of mental health is revised to break the negative consequences of social stigma (Haynes and Stroud, 2019). Many see social stigma about the nature of mental health difficulties as the single largest barrier to improvements across all aspects of public and health policy. Therefore, making extensive changes to pervasive social values, becomes the core component of building collective wellbeing resilience.

## Conclusion

The combining of complex systems theory with resilience results in a focus on the importance of a collective process that illuminates how resilience can be realised. It acknowledges that resilience as a complex social process is highly dynamic and subject to both disruptions and improvements. This is because the circumstances of resilience emerge primarily from relationships between people and collective systems of people, and the potential social transformations that these relationships can achieve.

The entanglement of values seen in complex social systems is also addressed in some of the more ecological orientated approaches to resilience. For example, Cameron et al. (2012) studied the return-to-work experiences of people with mental health problems. They concluded that individualising resilient responses which attempted to support people to ‘cope’ with work stresses were inadequate. Rather, there was a need to transform the adversity at work that contributed to poor mental health. This is where an employment culture is anti-resilient and preventing the social benefits of employment for welfare that are more usually anticipated. The focus becomes not the individual themselves, but working with them and similar others, and the social organisations they experience to alter their meso and macro environments. This transformation of the system environment in which resilience operates becomes a key element in the explanation that complexity theory brings to understandings of resilience. Positive system change includes an alignment with a set of public and social values. This change is not just static resilience to outside shocks but more likely a complete movement and adaptation of the system to a new and stable state. The World Health Organization’s (2021) vision of community based mental health services captures what such a system change can look like.

The authors’ resilience approach, (Hart et al., 2016) sees this as a social justice-oriented approach to resilience as, unlike the individualising and neoliberal interpretations, it involves challenging inequality rather than simply helping people to cope with its consequences. This process is intrinsically linked with the role of proactive public policy and the distribution of resources and not an alternative to them. This position represents a challenge to those critics of resilience (Haldane et al., 2017; Lockie, 2016) who have largely rejected the application of the concept of resilience to social practice claiming it amounts to putting a ‘sticking plaster’ on social and health problems.

Proponents of a social justice conceptualisation of resilience have not simply redefined resilience to ‘sidestep’ what can be understood as a form of a ‘structure versus agency’ debate between those who argue for macro change versus micro level coping. Rather, in reconceptualising adversity as part of a system dynamic they maintain a concern with *both* macro and micro level practice. This is a view of adversity that acknowledges that people often have immediate personal and cognitive needs to ‘cope’ with life challenges that cannot wait until major social reforms are made. It does not deny issues of structural inequality and a lack of social support, whose consequences can be best addressed by transforming society. Similarly, while Neocleous (2013) is concerned that resilience is the antithesis of resistance, we have argued that in some circumstances, resilience might be expressed as resistance. We acknowledge that resilience can be negatively impacted by social systems. Some system processes and adaptations can be anti-resilient. Manifestations of policy and market behaviours such as neo liberal globalisation and their impact on health and welfare services contribute to a decline in mental wellbeing. One way to achieve transformative change at multiple system levels is through designing and implementing comprehensive social models for mental health (World Health Organization, 2021). These will include changes in both, social values such as a reduction in stigma, alongside progressive and preventative developments in resource allocation and service provision (Reeve et al., 2016). Resilience building can be a consequence of such political and policy action rather than just a driver of it, although it can also play a part in such positive changes.

We argue the most important contribution of the combining of complex systems with resilience theory is that it leads to understanding a resilience system as a dynamic, social situation that occurs because of the formation of values and relationships. It is therefore the antithesis of individualism and an individual ‘becoming resilient’. It highlights the instability and conflicts of multiple social systems. Resilience is not a psychological state of mind. Resilience is the occurrence of a quality of supportive and collective social relations formed around shared values and approaches to adversity in unstable systems environments. This has major policy implications as it implies that to intervene in a way that ‘increases resilience’, is to intervene systemically and to build appropriate resource redistribution, shared values, open communication and quality relationships.

Lockie (2016: 116), argued: ‘the application of systems thinking and metaphors to the social realm is limited’, but we have argued that the grand narrative of complex systems and resilience theory offers a powerful theoretical framework for understanding the interactions, dynamics and indeterminable outcomes of the social realm. Fundamental to the adopting of complexity theory is not the dismantling of systems and resilience theory, but an acceptance of their mechanistic limitations. Applying complexity theory to resilience is therefore much more of a social process than an individual process. The societal arena of mental wellbeing clearly illustrates the need for a social perspective. The combination of resilience and complex systems approaches allows for a realistic and pragmatic theoretical framework that locates change and adaptation in the context of unstable and unpredictable social processes, collective action and the need for improvements to social justice.
